# Acute caffeine supplementation and cycling time-trial performance across different task durations: a systematic review and meta-analysis

**DOI:** 10.3389/fphys.2026.1853564

**Published:** 2026-06-29

**Authors:** Zhenyu Wang, Jingwen Zhang, Changmo Cho

**Affiliations:** 1 Department of Physical Education, Graduate School, Keimyung University, Daegu, Republic of Korea; 2 Department of Kinesiology, Keimyung University, Daegu, Republic of Korea

**Keywords:** caffeine, cycling performance, ergogenic aid, meta-analysis, time trial

## Abstract

**Background/objectives:**

Acute caffeine (CAF) ingestion may enhance cycling time-trial (TT) performance, but its ergogenic effects may be expressed differently across TT tasks with markedly different durations. Utilizing the power–duration relationship and critical power framework, this systematic review and meta-analysis examined the effects of acute pre-exercise CAF ingestion on cycling TT performance and compared effect estimates between prespecified short-duration (≤10 min) and long-duration (≥20 min) TT tasks.

**Methods:**

Following PRISMA 2020 guidelines and PROSPERO registration (CRD420261334904), five databases were searched from inception to 14 January 2026 for randomized, double-blind, placebo-controlled trials in healthy cyclists. Random-effects meta-analyses were conducted using standardized mean differences (SMDs; Hedges’ *g*) for TT completion time and mean power output.

**Results:**

Twenty-three trials were included. CAF reduced TT completion time (SMD = −0.32, 95% CI −0.49 to −0.16; *I*^2^ = 0%) and increased mean power output (SMD = 0.20, 95% CI 0.02 to 0.38; *I*^2^ = 0%). Completion time improved in both short-duration (SMD = −0.30) and long-duration (SMD = −0.37) TTs, with no significant subgroup difference. Mean power output increased significantly in short-duration TTs (SMD = 0.27) but did not reach statistical significance in long-duration TTs (SMD = 0.07), although the between-subgroup difference was not significant. Sensitivity analyses yielded consistent results.

**Conclusions:**

The findings of the present study indicate that acute pre-exercise caffeine ingestion can provide a small but practically meaningful benefit for cycling TT performance, primarily reflected in reduced completion time across the included prespecified short-duration (≤10 min) and long-duration (≥20 min) TT tasks. The increase in mean power output was mainly evident in short-duration TT tasks, suggesting that improved average mechanical power output may play an important role in short-duration performance gains, whereas improvements in long-duration TT tasks may involve additional performance-related factors beyond mean power output.

**Systematic Review Registration:**

https://www.crd.york.ac.uk/PROSPERO/view/CRD420261334904, identifier CRD420261334904.

## 1 Introduction

Caffeine (CAF) is one of the most widely studied and commonly used ergogenic aids in sports nutrition. Acute CAF supplementation is well documented to enhance various aspects of exercise performance, particularly aerobic endurance (Wang et al., 2022; Lowery et al., 2023). Proposed mechanisms include the antagonism of adenosine receptors within the central nervous system, reduced perceptions of effort and pain, enhanced neuromuscular activation, and potential alterations in substrate utilization during exercise (Bowtell et al., 2018; Cristina-Souza et al., 2022; Starling-Soares et al., 2023). In performance-based exercise tasks, these effects can translate into improved exercise tolerance, increased power output, delayed fatigue, and ultimately enhanced performance outcomes (Davis et al., 2003; Lima-Silva et al., 2021; Cristina-Souza et al., 2022; Lowery et al., 2023).

Cycling time trials (TTs) are typical performance-based tasks in which participants are required either to complete a set distance as quickly as possible or to maximize workload within a fixed duration (Jeukendrup et al., 1996). Among the available outcomes, completion time and mean power output constitute the most direct and practically relevant measures of cycling TT performance (Oliveira et al., 2017). Importantly, the physiological demands of TT tasks vary according to task duration (Thomas et al., 2015; Borszcz et al., 2018). Shorter TT tasks are generally performed at higher relative intensities and impose greater demands on anaerobic energy contributions, neuromuscular function, and the tolerance of high-intensity exercise (Thomas et al., 2015; Borszcz et al., 2018). In contrast, longer TT tasks rely primarily on aerobic metabolism, fatigue resistance, and sustained regulation of effort throughout the task (Thomas et al., 2015; Borszcz et al., 2018).

These duration-dependent differences suggest that the ergogenic effects of CAF may be expressed differently across cycling TT tasks of differing durations (Chen et al., 2024). In shorter TT tasks, CAF may exert its benefits primarily through enhanced central motor drive, increased neuromuscular recruitment, and attenuated perceived effort during high-intensity exercise (Felippe et al., 2018). Conversely, in longer TT tasks, CAF-related benefits may be more closely associated with reduced fatigue perception, improved alertness, and the sustained regulation of pacing and performance (Jaffe et al., 2018). Within the context of the power–duration relationship and the critical power (CP) framework, TT duration may therefore represent a physiologically relevant task characteristic for interpreting the performance-enhancing effects of CAF (Poole et al., 2016; Leo et al., 2022).

Several randomized, placebo-controlled trials have examined the effects of acute CAF ingestion on cycling TT performance. However, the findings remain inconsistent. Some studies have reported significant improvements in completion time and/or mean power output following CAF ingestion (Glaister et al., 2015; Santos et al., 2020), whereas others have failed to demonstrate significant benefits (Kilding et al., 2012; Bortolotti et al., 2014). These equivocal findings may be related to differences in participant characteristics, such as training status and habitual CAF intake, as well as variations in pre-trial CAF withdrawal, CAF dose and timing, and TT protocol parameters. Furthermore, such inconsistencies may partly reflect limited consideration of TT duration as a protocol-level factor influencing task demands (Poole et al., 2016; Leo et al., 2022).

Shen et al. previously examined the relationship between endurance TT event duration and the ergogenic effect of CAF, reporting a greater CAF effect size in longer-duration events (Shen et al., 2019). Although this review provided important duration-focused evidence, it synthesized endurance TT events across different exercise modalities and did not focus specifically on cycling TT performance or cycling-specific outcomes such as completion time and mean power output. Previous cycling-focused systematic reviews and meta-analyses have examined overall pooled effects and study-level factors such as dosage, training status, habitual CAF intake, age, and task duration (Chen et al., 2024; Wu et al., 2026). However, the comparison of effect estimates between prespecified short- and long-duration cycling TT tasks, informed by the power–duration relationship and critical power framework, has received less specific attention. As a result, cycling TT tasks with distinct physiological demands may be synthesized together, potentially limiting the task-specific interpretation and practical relevance of the findings.

In cycling TT contexts, comparing effect estimates between prespecified short- and long-duration TT tasks is also practically relevant for athletes, coaches, and sports nutrition practitioners. Such evidence may help inform CAF strategies tailored to different TT task demands and support more nuanced decision-making in applied sports nutrition.

Therefore, this systematic review and meta-analysis examined the effects of acute pre-exercise CAF ingestion on cycling TT performance, as reflected by completion time and mean power output. Utilizing the power–duration relationship and critical power framework, this study further compared effect estimates between prespecified short-duration (≤10 min) and long-duration (≥20 min) TT tasks. This duration-informed synthesis aimed to provide a more task-specific interpretation of the available cycling TT evidence.

## 2 Methods

### 2.1 Design

This systematic review and meta-analysis was conducted in accordance with the Preferred Reporting Items for Systematic Reviews and Meta-Analyses (PRISMA 2020) statement (Page et al., 2021). The review protocol was prospectively registered in the International Prospective Register of Systematic Reviews (PROSPERO; CRD420261334904).

### 2.2 Search strategy

A comprehensive literature search was conducted in PubMed, Embase, Cochrane Library, Web of Science, and Scopus from database inception to 14 January 2026. The search strategy employed a combination of free-text terms and database-specific controlled vocabularies, centered on the core concepts of “caffeine” and “cycling” (**Supplementary Table 1**). In addition, the reference lists of relevant systematic reviews and meta-analyses were manually screened to identify additional eligible studies.

### 2.3 Study selection

Following the removal of duplicates using EndNote 21, two reviewers (ZW and JZ) independently screened titles and abstracts and subsequently assessed the full texts of potentially eligible studies. Disagreements were resolved by discussion, with unresolved cases adjudicated by a third reviewer (CC).

### 2.4 Eligibility criteria

Eligibility was prespecified according to the PICOS framework: 1) population: healthy cyclists, including both trained/developmental and recreationally active individuals, with training status characterized according to McKay et al.’s participant classification framework based on training volume and performance caliber (McKay et al., 2022); 2) intervention: acute pre-exercise CAF ingestion administered as a single dose rather than chronic supplementation; 3) comparator: placebo or a control condition designed to isolate the independent effect of CAF; 4) outcomes: TT performance assessed using fixed-distance or fixed-time TT protocols, with TT completion time and/or mean power output reported; and 5) study design: randomized controlled trials, including crossover and parallel-group designs. Studies were excluded if they 1) involved clinical populations; 2) were non-human studies; 3) were not original, peer-reviewed full-text articles (e.g., reviews, study protocols, preprints, or conference abstracts); 4) did not employ a cycling TT protocol; 5) involved TT tasks within the intermediate duration range (>10 to <20 min); 6) included co-interventions that precluded isolation of the independent effect of CAF; or 7) were not published in English.

### 2.5 Data extraction

Two reviewers (ZW and JZ) independently extracted data from each included study and cross-checked all entries for accuracy. The extracted data included 1) study characteristics (first author, publication year, and country), 2) participant characteristics (sample size, sex, age, and training status), 3) intervention and comparator details (CAF dose, timing of ingestion, administration form, placebo/control condition, habitual CAF intake where available, and pre-trial CAF withdrawal), 4) study design and protocol characteristics (study design, blinding, and TT protocol characteristics, including task duration and/or distance), and 5) outcome data (TT completion time and/or mean power output).

### 2.6 Methodological quality assessment

Risk of bias was assessed using the Cochrane Risk of Bias tool (RoB1), and methodological quality was additionally assessed using the Physiotherapy Evidence Database (PEDro) scale (De Morton, 2009; Higgins et al., 2011). RoB1 assesses seven domains: random sequence generation, allocation concealment, blinding of participants and personnel, blinding of outcome assessment, incomplete outcome data, selective reporting, and other sources of bias. Each domain was rated as low, unclear, or high risk. For allocation concealment, studies were rated as low risk only when the method used to conceal allocation before assignment was explicitly described; when this information was insufficient or not reported, the domain was rated as unclear risk. The PEDro scale comprises 11 items, with the first item not contributing to the total score (range: 0–10). Studies were classified as poor (<4), fair (4–5), good (6–8), or excellent (≥9) (Zhang et al., 2022).

### 2.7 Certainty assessment

The Grading of Recommendations Assessment, Development and Evaluation (GRADE) approach was used to assess the certainty of evidence for the primary outcomes. The certainty was rated as high, moderate, low, or very low based on risk of bias, inconsistency, indirectness, imprecision, and publication bias. Two authors (ZW and JZ) independently performed the GRADE assessment, and disagreements were resolved by discussion with a third author (CC).

### 2.8 Statistical analysis

Means and standard deviations (SDs) for TT completion time and mean power output were extracted for the CAF and placebo conditions. Data were pooled using random-effects models to calculate standardized mean differences (SMDs; Hedges’ *g*) and 95% confidence intervals (CIs) (Higgins et al., 2019; Deeks et al., 2024). SMDs were used because TT protocols differed substantially in duration, distance, and absolute performance metrics across studies. For TT completion time, an SMD <0 indicated improved performance with CAF, whereas for mean power output, an SMD >0 indicated improved performance with CAF. When SDs were unavailable, they were derived from standard errors (SEs) or 95% CIs using established formulas (Deeks et al., 2024).

For crossover trials without reported paired-difference SDs or within-participant correlation coefficients, condition-specific data were analyzed as independent groups, yielding a conservative estimate of the treatment effect (Higgins et al., 2024). Statistical heterogeneity was assessed using Cochran’s *Q* test and the *I*^2^ statistic, with *I*^2^ values of approximately 25%, 50%, and 75% interpreted as low, moderate, and high heterogeneity, respectively (Deeks et al., 2024).

Subgroup analyses by TT duration were specified *a priori*. Duration categories were defined operationally to distinguish cycling TT protocols with clearly different task durations and expected physiological demands. This approach was informed by the power–duration relationship and critical power (CP) framework, which distinguish exercise performed above CP, where exercise tolerance is finite and work capacity above CP contributes substantially to performance, from longer efforts that are more strongly constrained by CP, maximal metabolic steady state, pacing regulation, and fatigue tolerance (Poole et al., 2016; Leo et al., 2022). Accordingly, TT tasks lasting ≤10 min were classified as short-duration tasks, as this range captures brief, high-intensity cycling TT protocols with greater non-steady-state demands and is consistent with empirical cycling studies that have examined short TT protocols of approximately 5–6 min alongside longer TT protocols (Thomas et al., 2015; Borszcz et al., 2018). TT tasks lasting ≥20 min were classified as long-duration tasks, as this range is commonly used to represent sustained endurance efforts and is relevant to performance near the critical power domain (Poole et al., 2016; Leo et al., 2022). For fixed-distance TT protocols, duration categories were assigned according to the reported or extractable mean completion time in the original studies. TT tasks lasting >10 to <20 min were not included in the primary duration-categorized subgroup analyses because this intermediate range may reflect transitional task demands and is therefore difficult to assign unambiguously to either duration category.

Differences between subgroups were assessed using the test for subgroup differences (Deeks et al., 2024). These subgroup analyses were designed as prespecified duration-categorized comparisons of effect estimates, with the interpretation limited to differences between the predefined short- and long-duration categories. Meta-regression analyses were not performed for study-level variables such as CAF dose, ingestion timing, age, training status, or habitual CAF intake, because of the limited number of available comparisons and incomplete or inconsistent reporting across studies. Accordingly, the duration-based analyses were limited to prespecified subgroup comparisons rather than adjusted moderator analyses.

Leave-one-out analyses were performed to assess the robustness of the pooled estimates. Forest plots were generated using Review Manager (RevMan, version 5.4), whereas sensitivity analyses, funnel plots, and Egger’s regression tests were conducted in R (version 4.5.1). Publication bias was interpreted cautiously when fewer than 10 comparisons were available (Afonso et al., 2024; Page et al., 2024). Statistical significance was set at *p <*0.05.

## 3 Results

### 3.1 Study selection

**Figure 1:**
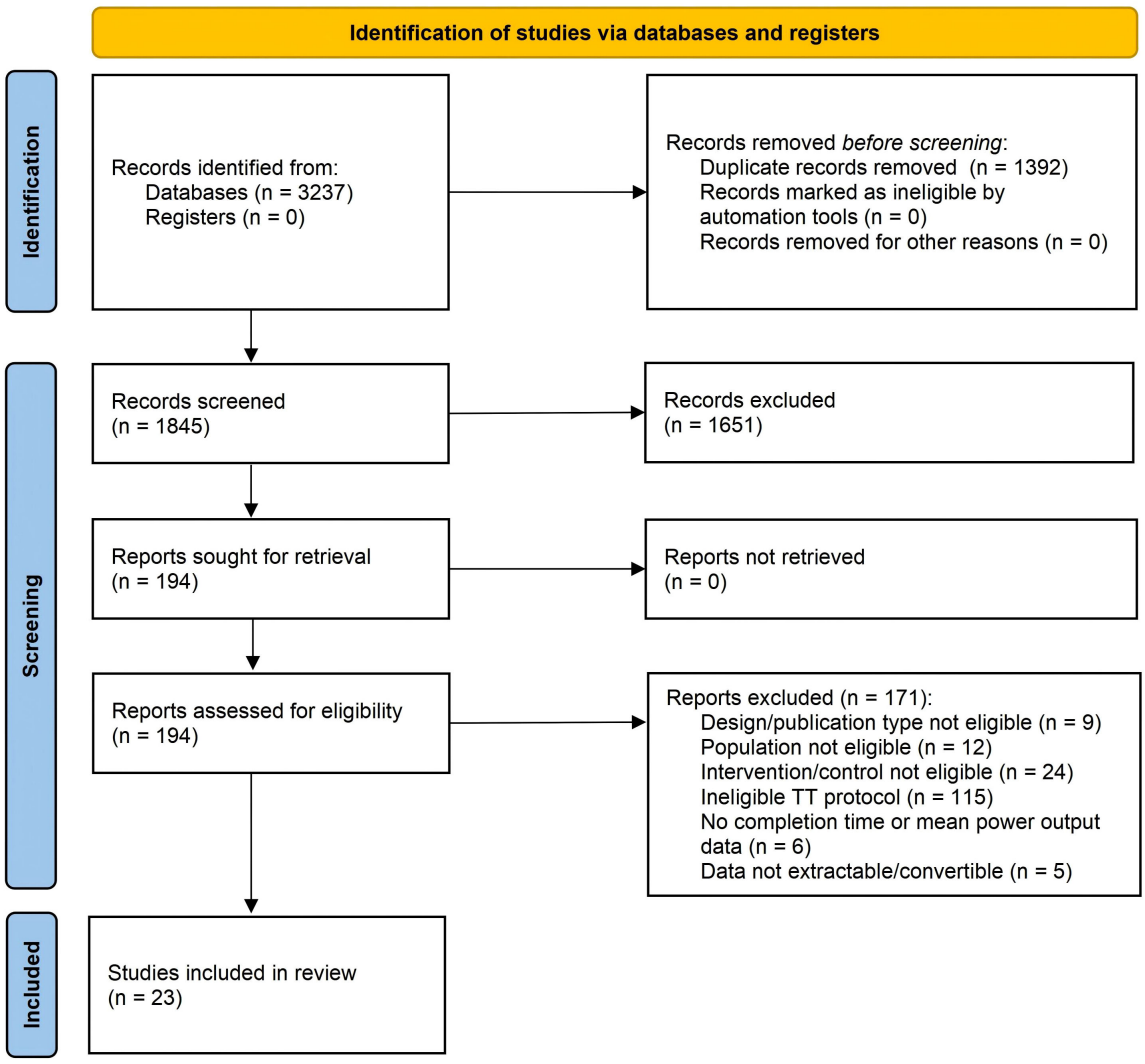
PRISMA flowchart of study selection.

As shown in **Figure 1**, the initial database search yielded 3,237 records (PubMed, *n* = 557; Web of Science, *n* = 864; Embase, *n* = 633; Cochrane Library, *n* = 673; Scopus, *n* = 510). After removal of duplicates, 1,845 records were screened by title and abstract, and 194 full-text articles were assessed for eligibility. Of these, 171 studies were excluded due to ineligible study design/publication type (*n* = 9), population (*n* = 12), intervention or control condition (*n* = 24), task type (*n* = 115), outcomes (*n* = 6), or non-extractable/non-convertible data (*n* = 5). Ultimately, 23 studies were included (Wiles et al., 2006; Acker-Hewitt et al., 2012; Kilding et al., 2012; Carneiro et al., 2013; Santos et al., 2013; Silva-Cavalcante et al., 2013; Skinner et al., 2013; Bortolotti et al., 2014; Glaister et al., 2015; Clarke et al., 2019; Ferreira Viana et al., 2020; Morales et al., 2020; Santos et al., 2020; Tomazini et al., 2020; Clarke and Richardson, 2021; Jessen et al., 2021; Couto et al., 2022; Tomazini et al., 2022; Marinho et al., 2024; Trujillo-Colmena et al., 2024; Sánchez-Redondo et al., 2025; Vinícius et al., 2025; Alejo et al., 2026).

### 3.2 Characteristics of included studies

The characteristics of the included studies and participants are summarized in **Table 1**. A total of 23 randomized, double-blind, placebo-controlled trials were included, all examining the effects of acute CAF ingestion on cycling TT performance.

**Table 1:** Characteristics of the studies included in this meta-analysis.

Study	Country	Participants	Age (yrs)	Training status	Habitual CAF intake	CAF protocol	TT task/duration category	Setting
Alejo et al., 2026	ES	13 NR	16.3 ± 0.5	Trained	NR	6 mg/kg, form NR, 60 min pre	Distance NR (~8 min, short)	Field
Acker-Hewitt et al., 2012	US	10 M	28 ± 9	Trained	NR	6 mg/kg, capsule, ~80 min pre	20 km (~49 min, long)	Lab
Bortolotti et al., 2014	BR	13 M	26 ± 10	Trained	NR	6 mg/kg, capsule, 60 min pre	20 km (~39 min, long)	Lab
Clarke et al., 2019	GB	19 M/19 F	M: 30 ± 5; F: 28 ± 6	Recreational	M: 191 ± 118; F: 214 ± 158	3 mg/kg, solution, 60 min pre	5 km (~9 min, short)	Lab
Couto et al., 2022	BR	9 M	32.3 ± 6	Trained	85.5 ± 71.3	5 mg/kg, capsule, 60 min pre	4 km (~6.5 min, short)	Lab
Carneiro et al., 2013	BR	10 M	27 ± 8	Trained	NR	6 mg/kg, capsule, 60 min pre	1 km (~1.5 min, short)	Lab
Clarke and Richardson, 2021	GB	27 M/19 F	M: 29 ± 6; F: 28 ± 6	Recreational	L: 153 ± 99; H: 415 ± 133	3 mg/kg, solution, 60 min pre	5 km (~8.3 min, short)	Lab
Ferreira Viana et al., 2020	BR	9 M	32 ± 7.5	Trained	50–250	6 mg/kg, capsule, 60 min pre	4 km (~6 min, short)	Lab
Glaister et al., 2015	GB	14 F	31 ± 7	Trained	249 ± 131	5 mg/kg, capsule, 60 min pre	20 km (~37 min, long)	Lab
Vinícius et al., 2025	NZ	13 M	36.2 ± 3.3	Trained	458.6 ± 375.5	5 mg/kg, capsule, 50 min pre	4 km (~6.5 min, short)	Lab
Jessen et al., 2021	DK	14 M	26 ± 6	Trained	NR	5 mg/kg, capsule, 60 min pre	Distance NR (~6 min, short)	Lab
Kilding et al., 2012	NZ	10 M	24.2 ± 5.4	Trained	NR	3 mg/kg, capsule, 60 min pre	3 km (~4 min, short)	Lab
Marinho et al., 2024	BR	10 M	24.7 ± 3.6	Recreational	75.44 ± 51.51	5 mg/kg, capsule, ~90 min pre	10 km (~22 min, long)	Lab
Morales et al., 2020	BR	14 M	34.1 ± 4.4	Trained	285.9 ± 108.0	6 mg/kg, capsule, 60 min pre	16 km (~30 min, long)	Lab
Santos et al., 2013	BR	8 M	32.6 ± 5.4	Trained	NR	5 mg/kg, capsule, 60 min pre	4 km (~7 min, short)	Lab
Silva-Cavalcante et al., 2013	BR	7 M	32.3 ± 5.4	Trained	NR	5 mg/kg, capsule, 60 min pre	4 km (~7 min, short)	Lab
Santos et al., 2020	BR	16 M	33.5 ± 5.2	Trained	NR	5 mg/kg, capsule, 60 min pre	4 km (~7 min, short)	Lab
Skinner et al., 2013	AU	14 M	31 ± 5.2	Trained	NR	6 mg/kg, capsule, 60 min pre	40 km (~59.1 min, long)	Lab
Sánchez-Redondo et al., 2025	ES	11 M	17 ± 1	Trained	39.5 ± 61.1	3 mg/kg, capsule, 60 min pre	6.4 km (~8 min, short)	Field
Tomazini et al., 2020	BR	11 M	24.5 ± 6.9	Recreational	93.3 ± 118.1	5 mg/kg, capsule, 60 min pre	4 km (~7 min, short)	Lab
Tomazini et al., 2022	BR	11 M	33 ± 7	Trained	171 ± 147	5 mg/kg, capsule, 50 min pre	4 km (~7 min, short)	Lab
Trujillo-Colmena et al., 2024	ES	9 M/2 F	22 ± 3	Recreational	0.79 ± 0.64 mg/kg/day	3 mg/kg, solution, 60 min pre	13.9 km (~50 min, long)	Field
Wiles et al., 2006	GB	8 M	32 ± 6	Trained	NR	5 mg/kg, solution, 60 min pre	1 km (~1.2 min, short)	Lab

Age is presented as mean ± SD where available. Habitual caffeine intake is reported as described in the original studies. Training status was interpreted with reference to McKay et al.’s participant classification framework, which considers training volume and performance caliber. “Short” TT tasks refer to tasks lasting ≤10 min, whereas “long” TT tasks refer to tasks lasting ≥20 min.

CAF, caffeine; TT, time trial; yrs, years; M, male; F, female; AU, Australia; NR, not reported; ES, Spain; US, United States; BR, Brazil; GB, Great Britain; NZ, New Zealand; DK, Denmark; pre, before exercise.

Most trials were conducted in laboratory or indoor settings, with three conducted in field or track settings (Trujillo-Colmena et al., 2024; Sánchez-Redondo et al., 2025; Alejo et al., 2026). Overall, the evidence base was predominantly male: 18 studies enrolled men only (Wiles et al., 2006; Acker-Hewitt et al., 2012; Kilding et al., 2012; Carneiro et al., 2013; Santos et al., 2013; Silva-Cavalcante et al., 2013; Skinner et al., 2013; Bortolotti et al., 2014; Ferreira Viana et al., 2020; Morales et al., 2020; Santos et al., 2020; Tomazini et al., 2020; Jessen et al., 2021; Couto et al., 2022; Tomazini et al., 2022; Marinho et al., 2024; Sánchez-Redondo et al., 2025; Vinícius et al., 2025), one enrolled women only (Glaister et al., 2015), three enrolled both sexes (Clarke et al., 2019; Clarke and Richardson, 2021; Trujillo-Colmena et al., 2024), and one did not report participant sex (Alejo et al., 2026). By training status, 18 studies included trained cyclists (recreationally trained and/or competitive) (Wiles et al., 2006; Acker-Hewitt et al., 2012; Kilding et al., 2012; Carneiro et al., 2013; Santos et al., 2013; Silva-Cavalcante et al., 2013; Skinner et al., 2013; Bortolotti et al., 2014; Glaister et al., 2015; Ferreira Viana et al., 2020; Morales et al., 2020; Santos et al., 2020; Jessen et al., 2021; Couto et al., 2022; Tomazini et al., 2022; Sánchez-Redondo et al., 2025; Vinícius et al., 2025; Alejo et al., 2026), whereas five included recreational or non-trained cyclists (Clarke et al., 2019; Tomazini et al., 2020; Clarke and Richardson, 2021; Marinho et al., 2024; Trujillo-Colmena et al., 2024). Habitual CAF intake was reported in 12 studies (Glaister et al., 2015; Clarke et al., 2019; Ferreira Viana et al., 2020; Morales et al., 2020; Tomazini et al., 2020; Clarke and Richardson, 2021; Couto et al., 2022; Tomazini et al., 2022; Marinho et al., 2024; Trujillo-Colmena et al., 2024; Sánchez-Redondo et al., 2025; Vinícius et al., 2025) but not in 11 studies (Wiles et al., 2006; Acker-Hewitt et al., 2012; Kilding et al., 2012; Carneiro et al., 2013; Santos et al., 2013; Silva-Cavalcante et al., 2013; Skinner et al., 2013; Bortolotti et al., 2014; Santos et al., 2020; Jessen et al., 2021; Alejo et al., 2026). Regarding TT protocols, 16 studies were classified as short-duration TT (Wiles et al., 2006; Kilding et al., 2012; Carneiro et al., 2013; Santos et al., 2013; Silva-Cavalcante et al., 2013; Clarke et al., 2019; Ferreira Viana et al., 2020; Santos et al., 2020; Tomazini et al., 2020; Clarke and Richardson, 2021; Jessen et al., 2021; Couto et al., 2022; Tomazini et al., 2022; Sánchez-Redondo et al., 2025; Vinícius et al., 2025; Alejo et al., 2026) and seven as long-duration TT (Acker-Hewitt et al., 2012; Skinner et al., 2013; Bortolotti et al., 2014; Glaister et al., 2015; Morales et al., 2020; Marinho et al., 2024; Trujillo-Colmena et al., 2024). For pre-test controls, 21 studies implemented CAF withdrawal before testing (Wiles et al., 2006; Acker-Hewitt et al., 2012; Kilding et al., 2012; Carneiro et al., 2013; Santos et al., 2013; Silva-Cavalcante et al., 2013; Skinner et al., 2013; Bortolotti et al., 2014; Glaister et al., 2015; Clarke et al., 2019; Ferreira Viana et al., 2020; Morales et al., 2020; Santos et al., 2020; Tomazini et al., 2020; Clarke and Richardson, 2021; Jessen et al., 2021; Couto et al., 2022; Tomazini et al., 2022; Marinho et al., 2024; Trujillo-Colmena et al., 2024; Sánchez-Redondo et al., 2025; Alejo et al., 2026), whereas two did not report withdrawal procedures (Vinícius et al., 2025; Alejo et al., 2026).

CAF ingestion was generally scheduled before the start of the exercise protocol. In most studies, CAF was administered approximately 60 min before the start of the exercise protocol (Wiles et al., 2006; Kilding et al., 2012; Carneiro et al., 2013; Santos et al., 2013; Silva-Cavalcante et al., 2013; Skinner et al., 2013; Bortolotti et al., 2014; Glaister et al., 2015; Clarke et al., 2019; Ferreira Viana et al., 2020; Morales et al., 2020; Santos et al., 2020; Tomazini et al., 2020; Clarke and Richardson, 2021; Jessen et al., 2021; Couto et al., 2022; Trujillo-Colmena et al., 2024; Sánchez-Redondo et al., 2025; Alejo et al., 2026), while two studies administered CAF 50 min before exercise (Tomazini et al., 2022; Vinícius et al., 2025). In studies with a pre-TT exercise phase, the interval between CAF ingestion and TT start was longer, including approximately 80 min in Acker-Hewitt et al. (2012) and approximately 90 min in Marinho et al. (2024).

### 3.3 Risk of bias

Using the Cochrane Risk of Bias tool (RoB1), no study was judged to be at high risk of bias in any domain. Overall, risk of bias was generally low across domains. In accordance with the predefined criterion, unclear ratings occurred mainly because allocation concealment was insufficiently reported (*n* = 14) (Wiles et al., 2006; Acker-Hewitt et al., 2012; Santos et al., 2013; Skinner et al., 2013; Bortolotti et al., 2014; Glaister et al., 2015; Clarke et al., 2019; Ferreira Viana et al., 2020; Santos et al., 2020; Tomazini et al., 2020; Couto et al., 2022; Tomazini et al., 2022; Marinho et al., 2024; Trujillo-Colmena et al., 2024). Only a small number of studies were rated as unclear for blinding of participants/personnel (*n* = 1) (Wiles et al., 2006), incomplete outcome data (*n* = 1) (Santos et al., 2020), selective reporting (*n* = 1) (Santos et al., 2020), or other bias (*n* = 1) (Kilding et al., 2012) (**Supplementary Figure 1**, **S2**). Consistent with these findings, PEDro appraisal indicated that most studies were of excellent methodological quality (*n* = 18) (Kilding et al., 2012; Santos et al., 2013; Silva-Cavalcante et al., 2013; Skinner et al., 2013; Bortolotti et al., 2014; Glaister et al., 2015; Clarke et al., 2019; Ferreira Viana et al., 2020; Morales et al., 2020; Tomazini et al., 2020; Clarke and Richardson, 2021; Couto et al., 2022; Tomazini et al., 2022; Marinho et al., 2024; Trujillo-Colmena et al., 2024; Sánchez-Redondo et al., 2025; Vinícius et al., 2025; Alejo et al., 2026), with the remaining studies rated as good quality (*n* = 5) (Wiles et al., 2006; Acker-Hewitt et al., 2012; Carneiro et al., 2013; Santos et al., 2020; Jessen et al., 2021) (**Supplementary Table 3**).

### 3.4 Meta-analysis

#### 3.4.1 Time-trial completion time

**Figure 2:**
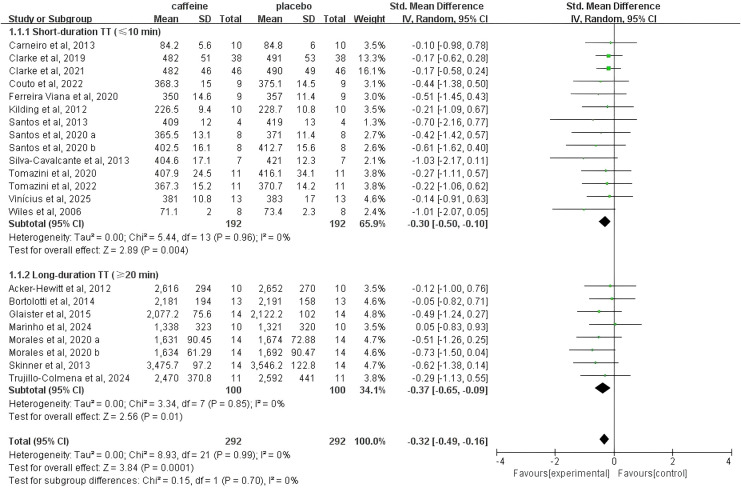
Forest plot of the effects of acute CAF ingestion on cycling TT completion time (random-effects model), overall and by duration (≤10 vs. ≥20 min). SMDs with 95% CIs are shown.

Across 22 comparisons, the analysis included 292 observations in the CAF condition and 292 observations in the placebo condition. A random-effects meta-analysis showed that acute CAF ingestion significantly reduced cycling TT completion time (SMD = −0.32, 95% CI −0.49 to −0.16; *p* = 0.0001), with no heterogeneity (*I*^2^ = 0%; **Figure 2**). In the prespecified duration-categorized subgroup analyses, significant improvements were observed for both short-duration TT (≤10 min; SMD = −0.30, 95% CI −0.50 to −0.10; *p* = 0.004; *I*^2^ = 0%) and long-duration TT (≥20 min; SMD = −0.37, 95% CI −0.65 to −0.09; *p* = 0.01; *I*^2^ = 0%). There was no evidence of a subgroup difference (*p* = 0.70; **Figure 2**), indicating that the improvement in TT completion time was broadly comparable between the two prespecified duration categories.

#### 3.4.2 Mean power output

**Figure 3:**
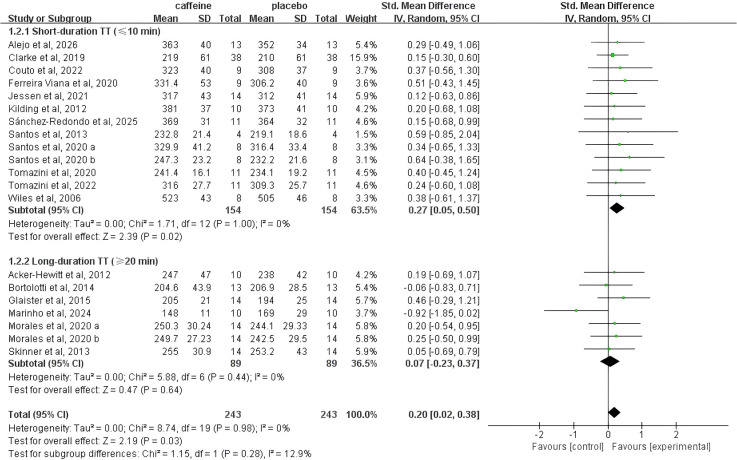
Forest plot of the effects of acute CAF ingestion on mean power output during cycling TT (random-effects model), overall and by duration (≤10 vs. ≥20 min). SMDs with 95% CIs are shown.

Across 20 comparisons, the analysis included 243 observations in the CAF condition and 243 observations in the placebo condition. A random-effects meta-analysis showed that acute CAF ingestion significantly increased mean power output during cycling TT (SMD = 0.20, 95% CI 0.02 to 0.38; *p* = 0.03), with no statistical heterogeneity (*I*^2^ = 0%; **Figure 3**). In the prespecified duration-categorized subgroup analyses, a significant increase was observed in short-duration TT (≤10 min; SMD = 0.27, 95% CI 0.05 to 0.50; *p* = 0.02; *I*^2^ = 0%), whereas the effect was not significant in long-duration TT (≥20 min; SMD = 0.07, 95% CI −0.23 to 0.37; *p* = 0.64; *I*^2^ = 0%). There was no evidence of a subgroup difference (*p* for subgroup difference = 0.28; **Figure 3**).

### 3.5 Publication bias

Visual inspection of the funnel plots did not suggest marked asymmetry for either outcome, overall, or within prespecified duration-categorized subgroup analyses (**Supplementary Figure 3–8**). Consistent with this observation, Egger’s regression tests showed no evidence of small-study effects for TT completion time (overall: *p* = 0.133; short-duration: *p* = 0.103; long-duration: *p* = 0.223) or mean power output (overall: *p* = 0.588; short-duration: *p* = 0.340; long-duration: *p* = 0.083). These findings should nevertheless be interpreted cautiously, particularly in subgroup analyses with a limited number of studies.

### 3.6 Sensitivity analysis

Leave-one-out sensitivity analyses showed that exclusion of any single study did not materially alter the pooled estimates for TT completion time or mean power output (**Supplementary Figures 9–14**). The direction and overall interpretation of the results remained unchanged. Similar patterns were observed in the prespecified duration-categorized subgroup analyses (≤10 and ≥20 min), indicating that no individual study had an undue influence on the pooled estimates.

### 3.7 GRADE summary

Using the GRADE approach, the certainty of evidence was rated as moderate for both primary outcomes. The evidence was downgraded by one level for risk of bias, mainly because allocation concealment was frequently unclear due to incomplete reporting across the included trials. This appears to reflect a common reporting limitation of acute exercise–nutrition crossover randomized trials rather than clear evidence of systematically flawed methods. No serious concerns were identified for inconsistency, indirectness, imprecision, or publication bias.

## 4 Discussion

### 4.1 Main findings

The primary objective of this systematic review and meta-analysis was to evaluate the overall ergogenic effects of acute pre-exercise CAF ingestion on cycling TT performance. Utilizing the power–duration relationship and critical power framework, we further compared effect estimates between prespecified short-duration (≤10 min) and long-duration (≥20 min) TT tasks. The main finding was that acute CAF ingestion produced a small but statistically significant improvement in cycling TT performance, as reflected by reduced completion time and increased mean power output. From a practical perspective, the pooled SMDs observed for completion time (SMD = −0.32) and mean power output (SMD = 0.20) indicate small, rather than large, performance improvements, based on conventional benchmarks for standardized effect sizes (Cohen, 2013). Nevertheless, even small improvements in completion time or mean power output may be meaningful in competitive cycling, where performance margins are often narrow.

In the duration-categorized subgroup analyses, CAF significantly reduced completion time in both short-duration and long-duration TT tasks, with no evidence of a between-subgroup difference. For mean power output, CAF resulted in an improvement for short-duration TT, but not for long-duration TT. Importantly, the between-subgroup difference indicated statistical similarity. Therefore, these subgroup findings should be interpreted cautiously as comparisons between prespecified duration categories should not be taken as definitive evidence that TT duration modifies the ergogenic effect of CAF.

Although statistical heterogeneity was negligible across the main analyses, this result also requires careful interpretation. An *I*^2^ value of 0% indicates that no between-study heterogeneity was detected, but it does not imply that the included studies were clinically or methodologically homogeneous. The included trials differed in participants’ training status, habitual CAF intake, pre-trial CAF withdrawal procedures, CAF dose, administration form, and TT protocol characteristics. Accordingly, the absence of statistical heterogeneity should be interpreted alongside the observed variability across the included studies.

### 4.2 Acute CAF supplementation and cycling TT completion time

The present systematic review and meta-analysis showed that acute pre-exercise CAF ingestion significantly reduced cycling TT completion time, suggesting a small ergogenic benefit that may be practically relevant. This finding is broadly consistent with previous evidence supporting the efficacy of CAF as an ergogenic aid for endurance exercise performance (Trujillo-Colmena et al., 2024). When trials were stratified according to the prespecified duration categories, CAF significantly reduced completion time in both short-duration (≤10 min) and long-duration (≥20 min) TT tasks, with no evidence of a between-subgroup difference. Collectively, these findings suggest that the benefit of CAF for TT completion time was observed across both duration categories.

Mechanistically, the improvement in completion time may reflect CAF’s influence on fatigue perception, alertness, and effort regulation during self-paced exercise (Cristina-Souza et al., 2022; Lowery et al., 2023). From a psychobiological perspective, these effects may help cyclists sustain goal-directed effort despite a higher perceptual cost during self-paced TT performance. This interpretation is also consistent with central fatigue perspectives, which emphasize central regulation of perceived exertion, fatigue perception, and voluntary motor drive during endurance performance. As a non-selective adenosine receptor antagonist, CAF may attenuate perceptions of fatigue and exertion, increase central drive, and support neuromuscular function (Meeusen et al., 2013; Pereira et al., 2016; Barceló Cormano et al., 2019). In TT tasks, these effects may not necessarily manifest as marked changes in a single physiological variable. Instead, they may contribute to improved pacing decisions, a more favorable distribution of effort, and better maintenance of performance late in the task, ultimately leading to faster completion time (Rosales Soto et al., 2015; Mitchell and Podczerwinski, 2017). Accordingly, completion time represents an ecologically valid endpoint that integrates both physiological and perceptual contributions to TT performance.

Although significant benefits were observed in both duration subgroups, the mechanisms contributing to improved completion time may vary according to task demands (Lima-Silva et al., 2021; Stadheim et al., 2021). Shorter TT tasks are typically performed at higher relative intensities and rely more heavily on high-intensity power production, neuromuscular recruitment, and tolerance of exercise-induced discomfort (Froyd et al., 2020). Under these conditions, CAF-related benefits may partly reflect enhanced central motor drive and reduced perceived exertion (Santos Amador et al., 2025). In contrast, longer TT tasks rely more on fatigue regulation, pacing control, and the sustained maintenance of performance. In this context, CAF’s benefits may be more closely associated with preserved alertness, reduced fatigue perception, and more effective pacing regulation (Chen et al., 2024). Within the power–duration and CP framework, task duration provides a useful physiological context for interpreting the demands of TT tasks and the possible mechanisms through which CAF may contribute to improved completion time (Poole et al., 2016; Leo et al., 2022). This interpretation aligns with the present observation of similar benefits in completion time across duration categories.

Importantly, the absence of a significant subgroup difference should not be interpreted as evidence of identical responses across TT tasks; it suggests that current evidence is insufficient to establish TT duration as a reliable effect modifier for completion time. The relatively consistent timing of CAF ingestion across most included studies may also have reduced variability related to absorption timing (Hayat et al., 2022). Overall, the available evidence supports acute CAF supplementation as a practical pre-competition strategy for improving cycling TT completion time in the included short- and long-duration TT tasks, although the subgroup findings should be interpreted within the limits of the prespecified duration categories.

### 4.3 Acute CAF supplementation and mean power output during cycling TT

This systematic review and meta-analysis indicated that acute pre-exercise CAF ingestion increased mean power output during cycling TT. In the prespecified duration-categorized analyses, the findings were not fully consistent between short- and long-duration TT tasks. Subgroup analyses showed a significant increase in mean power output for short-duration (≤10 min) TT tasks, whereas the pooled effect estimate for long-duration (≥20 min) TT tasks was not statistically significant. However, the between-subgroup comparison was also not statistically significant. Collectively, these findings suggest a possible short-duration pattern for mean power output, although the available evidence remains insufficient to establish TT duration as a clear effect modifier. In contrast to completion time, mean power output provides a more direct representation of the capacity to sustain an external work rate throughout the TT and is closely related to pacing strategy, fatigue tolerance, and effort distribution (Atkinson et al., 2007; Aisbett et al., 2015). Consequently, CAF-related reductions in perceived exertion and fatigue, together with improved alertness and central motor drive, may be reflected in mean power output (Doherty et al., 2004; Hodgson et al., 2013). Accordingly, mean power output may capture aspects of CAF’s ergogenic effect that are not fully reflected by completion time, particularly in self-paced tasks in which athletes continuously regulate their power output.

Although the pooled effect was statistically significant only in short-duration TT tasks, the mechanisms contributing to changes in mean power output may vary according to task demands (Kraemer and Looney, 2012; Goulding and Marwood, 2023). Short-duration TT tasks typically require greater high-intensity power maintenance and neuromuscular recruitment. Under these conditions, CAF-related effects on central drive and fatigue tolerance may be reflected in increased mean power output (Bowtell et al., 2018; Cristina-Souza et al., 2022; Starling-Soares et al., 2023). In longer TT tasks, performance is strongly influenced by pacing regulation and cumulative fatigue, potentially making small treatment effects more difficult to detect (Skorski et al., 2015; Binkley et al., 2021; Voet et al., 2024). Although statistical heterogeneity was low across both duration subgroups (*I*^2^ = 0%), the non-significant pooled effect in long-duration TT tasks should be interpreted cautiously. This finding may reflect limited subgroup sample size, reduced statistical precision, or protocol variation not fully captured by *I*^2^. Within the power–duration and CP framework, short-duration TT tasks place greater emphasis on high-intensity capacity, whereas longer TT tasks place greater emphasis on sustained effort regulation and fatigue management (Wiles et al., 2006; Bortolotti et al., 2014). Accordingly, CAF-related effects may be reflected differently in mean power output between these prespecified duration categories.

Similarly, the absence of a significant subgroup difference should not be interpreted as evidence of identical performance responses between short- and long-duration TT tasks. Given the limited number of available studies and the relatively small sample sizes within each subgroup, the statistical power to detect true subgroup effects was likely limited (Boyett et al., 2016; Cuijpers et al., 2021; Chen et al., 2024). Collectively, these findings suggest that completion time and mean power output may reflect CAF’s ergogenic action differently across the prespecified duration categories, without providing definitive evidence that TT duration modifies the overall effect of CAF. In summary, acute CAF supplementation appears to increase mean power output during cycling TT, but the apparent short-duration pattern should be interpreted cautiously given the non-significant subgroup difference.

### 4.4 Limitations

Several limitations should be acknowledged. First, although the duration-categorized subgroup analyses were prespecified, the number of studies and the sample sizes within each subgroup were limited, reducing the statistical power to detect true subgroup differences. Second, the study samples were predominantly male, which limits the generalizability of the findings across sexes. Third, despite low statistical heterogeneity in the main analyses, the included studies varied in training status, habitual CAF intake, pre-trial CAF withdrawal procedures, CAF dose, administration form, and TT protocol characteristics, which may still have influenced the magnitude of the observed ergogenic effect. The limited number of available comparisons and incomplete reporting across studies also restricted the ability to evaluate these potential sources of variation in greater detail. Fourth, TT tasks of intermediate duration (>10 to <20 min) were excluded to maintain clearer separation between short- and long-duration categories, which limits the applicability of the findings to this intermediate-duration range and reduces the completeness of the evidence base. Accordingly, the present findings should be interpreted as a duration-categorized synthesis of prespecified short- and long-duration TT tasks, and not as definitive evidence that TT duration independently modifies the ergogenic effect of CAF. Finally, for some crossover trials, paired-effect information was unavailable, and these trials were analyzed as independent-group comparisons, which may have reduced statistical precision and influenced the weighting of these comparisons.

## 5 Conclusions

The findings of the present meta-analysis indicate that acute pre-exercise caffeine ingestion may provide a small but practically meaningful benefit for cycling TT performance, primarily reflected in reduced completion time across the included prespecified short-duration (≤10 min) and long-duration (≥20 min) TT tasks. The increase in mean power output was mainly evident in short-duration TT tasks, suggesting that improved average mechanical power output may play an important role in short-duration performance gains, whereas improvements in long-duration TT tasks may involve additional performance-related factors beyond mean power output. Although the magnitude of caffeine’s ergogenic effect was modest, such marginal improvements may retain practical relevance in competitive cycling, where small differences in completion time can be decisive. Future research should further clarify the physiological and perceptual mechanisms through which caffeine may enhance performance in long-duration cycling tasks, particularly when mean power output does not increase significantly.

## Data Availability

The original contributions presented in the study are included in the article/**Supplementary Material**. Further inquiries can be directed to the corresponding author.
