# Supplementary material for: Acute caffeine supplementation and cycling time-trial performance across different task durations: a systematic review and meta-analysis
Source: Front Physiol. 2026 Jun 29;17:1853564. doi: 10.3389/fphys.2026.1853564 (PMC13357162; doi:10.3389/fphys.2026.1853564)
Supplement: Supplementary file 1 [file SupplementaryFile1.docx]

Acute Caffeine Supplementation and Cycling Time-Trial Performance Across Different Task Durations: A Systematic Review and Meta-Analysis

[Figure S1. Risk-of-bias summary of the included studies based on the Cochrane risk of bias tool. 1](#_Toc1182)

[Figure S2. Risk-of-bias graph of the included studies based on the Cochrane risk of bias tool. 1](#_Toc27251)

[Figure S3. Funnel plot of completion time (overall). 2](#_Toc3831)

[Figure S4. Funnel plot of completion time (short-duration TT, ≤10 min) 2](#_Toc26831)

[Figure S5. Funnel plot of completion time (long-duration TT, ≥20 min). 3](#_Toc26824)

[Figure S6. Funnel plot of mean power output (overall). 3](#_Toc21837)

[Figure S7. Funnel plot of mean power output (short-duration TT, ≤10 min). 4](#_Toc19361)

[Figure S8. Funnel plot of mean power output (long-duration TT, ≥20 min). 4](#_Toc29033)

[Figure S9. Leave-one-out sensitivity analysis of completion time (overall). 5](#_Toc28792)

[Figure S10. Leave-one-out sensitivity analysis of completion time (short-duration TT, ≤10 min). 5](#_Toc16901)

[Figure S11. Leave-one-out sensitivity analysis of completion time (long-duration TT, ≥20 min). 6](#_Toc16734)

[Figure S12. Leave-one-out sensitivity analysis of mean power output (overall). 6](#_Toc1729)

[Figure S13. Leave-one-out sensitivity analysis of mean power output (short-duration TT, ≤10 min). 7](#_Toc19971)

[Figure S14. Leave-one-out sensitivity analysis of mean power output (long-duration TT, ≥20 min). 7](#_Toc19694)

[Table S1. Database Search Details 8](#_Toc16712)

[Table S2. Characteristics of the studies included in this meta-analysis. 9](#_Toc859)

[Table S3. PEDro scores of the included studies. 14](#_Toc18540)

[Table S4. PRISMA 2020 checklist. 15](#_Toc23885)


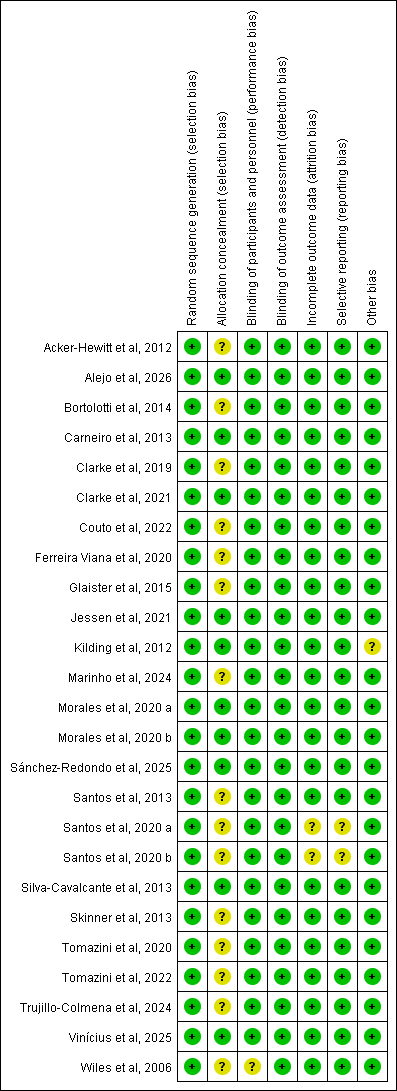


# Figure S1. Risk-of-bias summary of the included studies based on the Cochrane risk of bias tool.


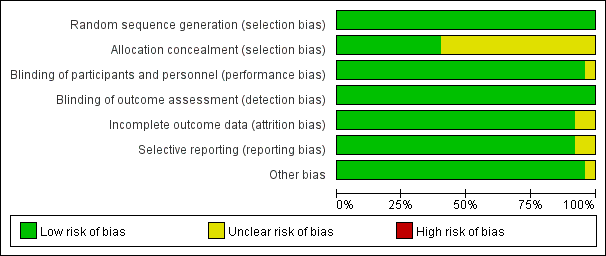


# Figure S2. Risk-of-bias graph of the included studies based on the Cochrane risk of bias tool.


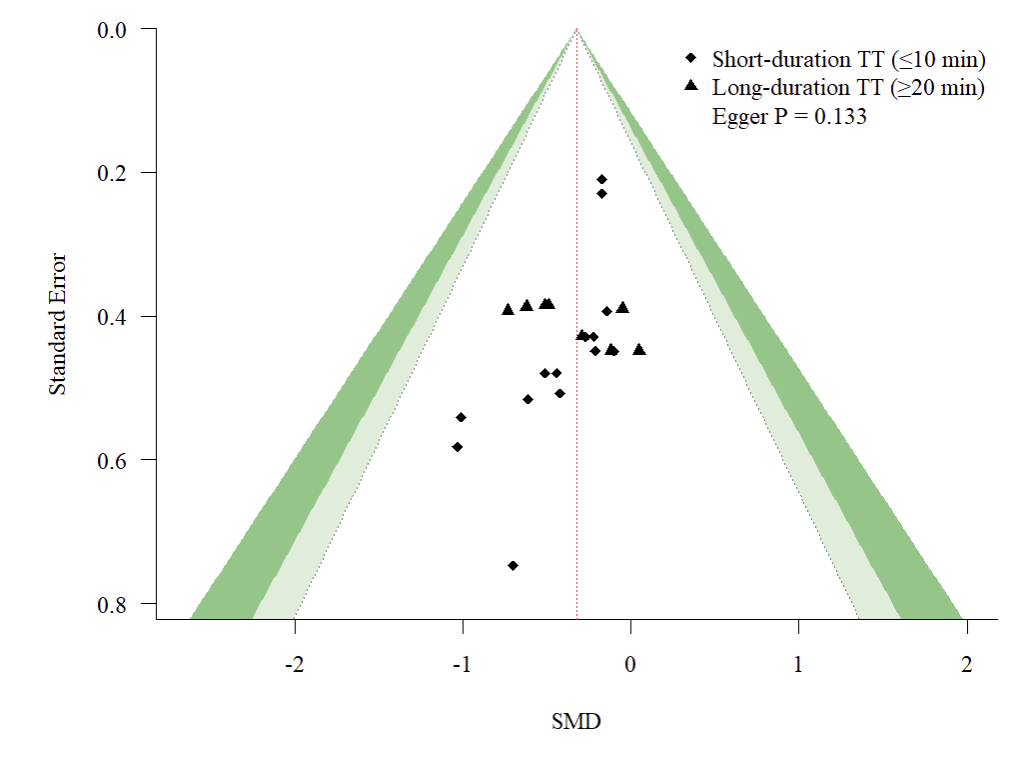


# Figure S3. Funnel plot of completion time (overall).


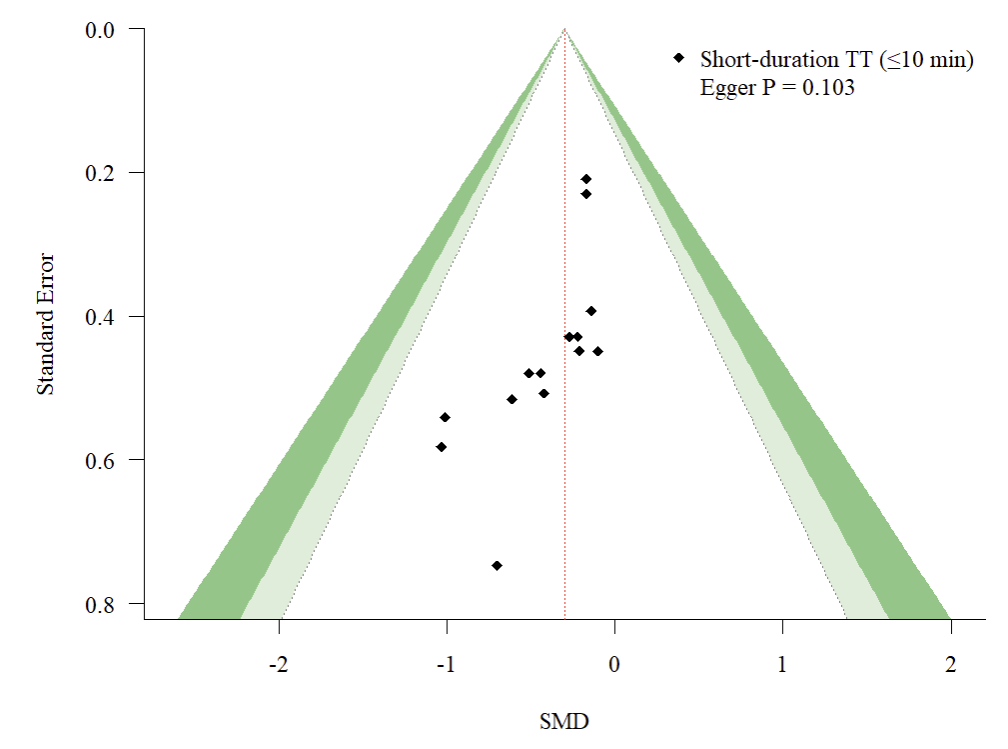


# Figure S4. Funnel plot of completion time (short-duration TT, ≤10 min)


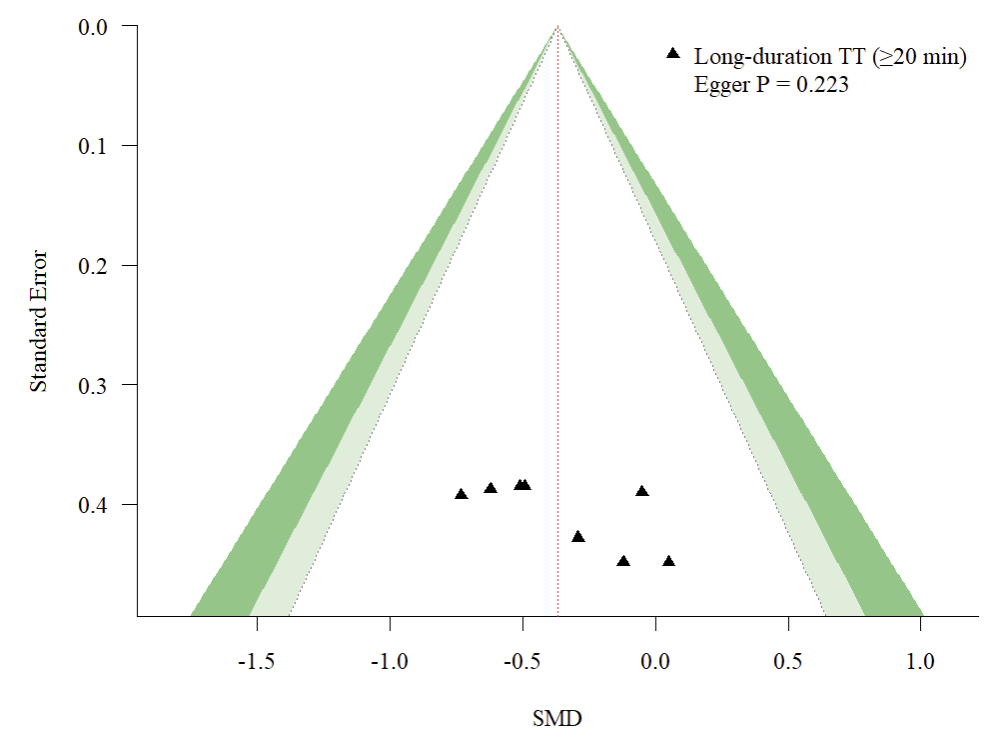


# Figure S5. Funnel plot of completion time (long-duration TT, ≥20 min).


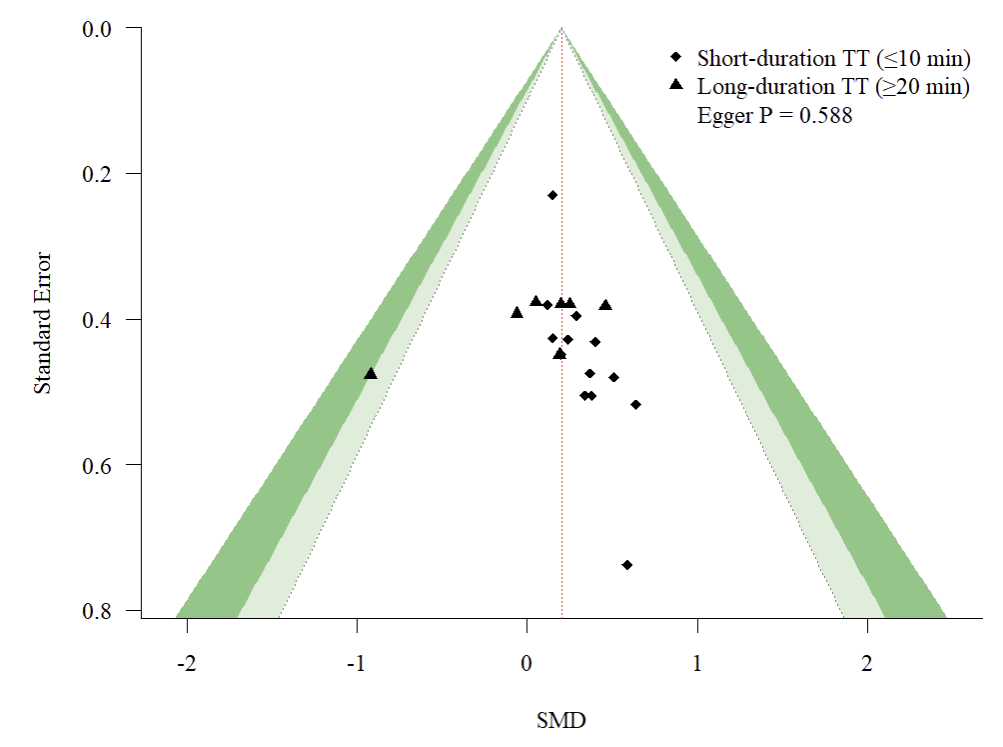


# Figure S6. Funnel plot of mean power output (overall).


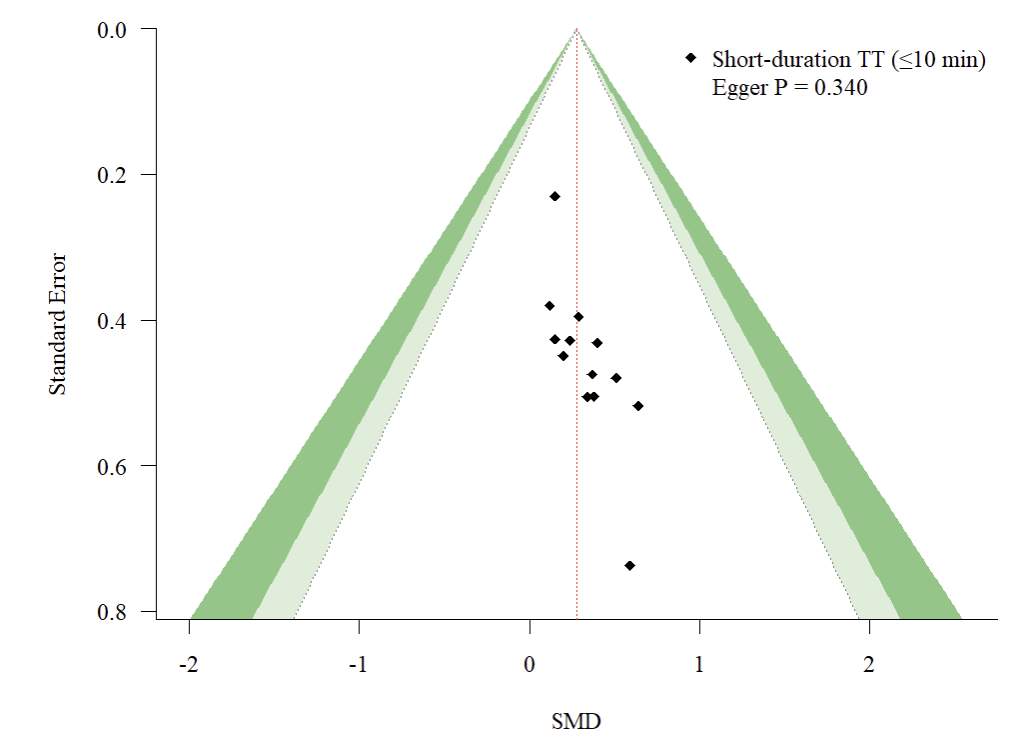


# Figure S7. Funnel plot of mean power output (short-duration TT, ≤10 min).


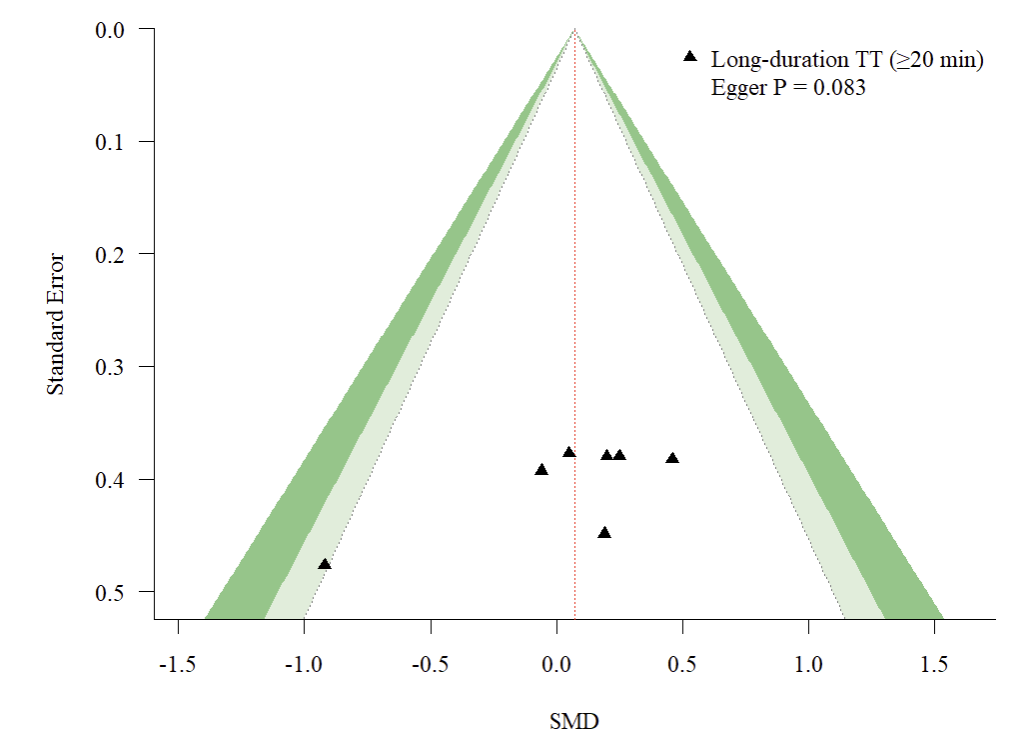


# Figure S8. Funnel plot of mean power output (long-duration TT, ≥20 min).


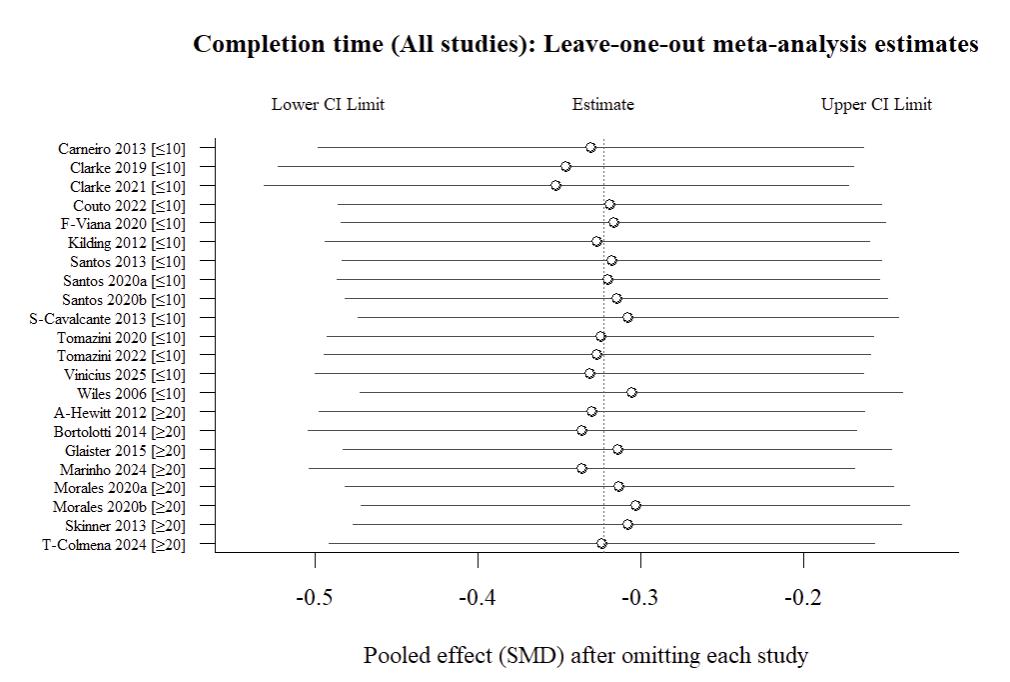


# Figure S9. Leave-one-out sensitivity analysis of completion time (overall).


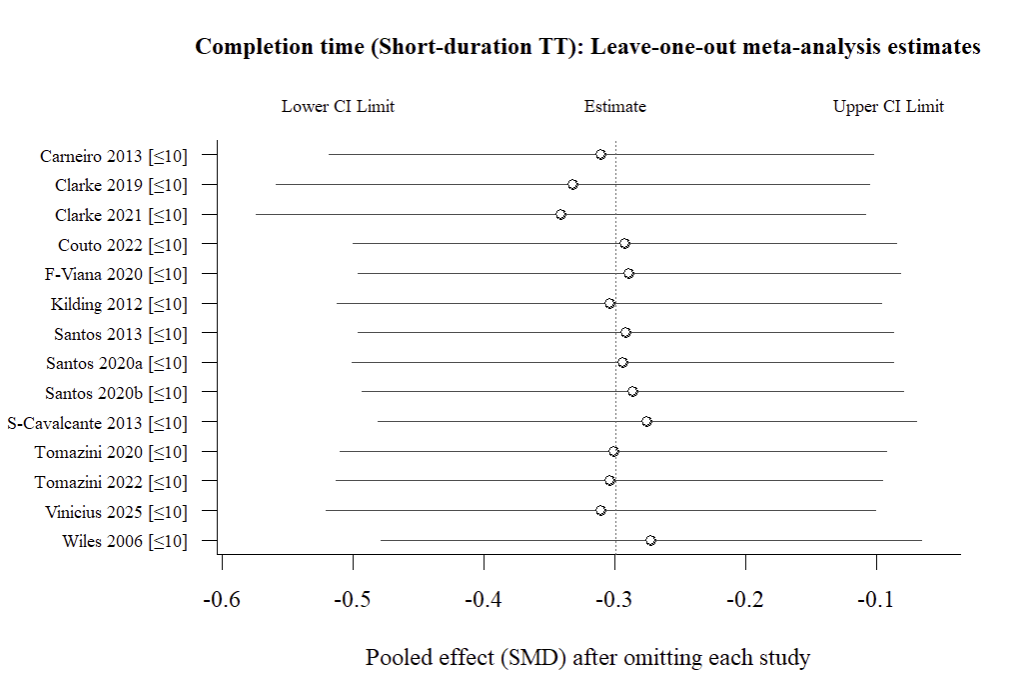


# Figure S10. Leave-one-out sensitivity analysis of completion time (short-duration TT, ≤10 min).


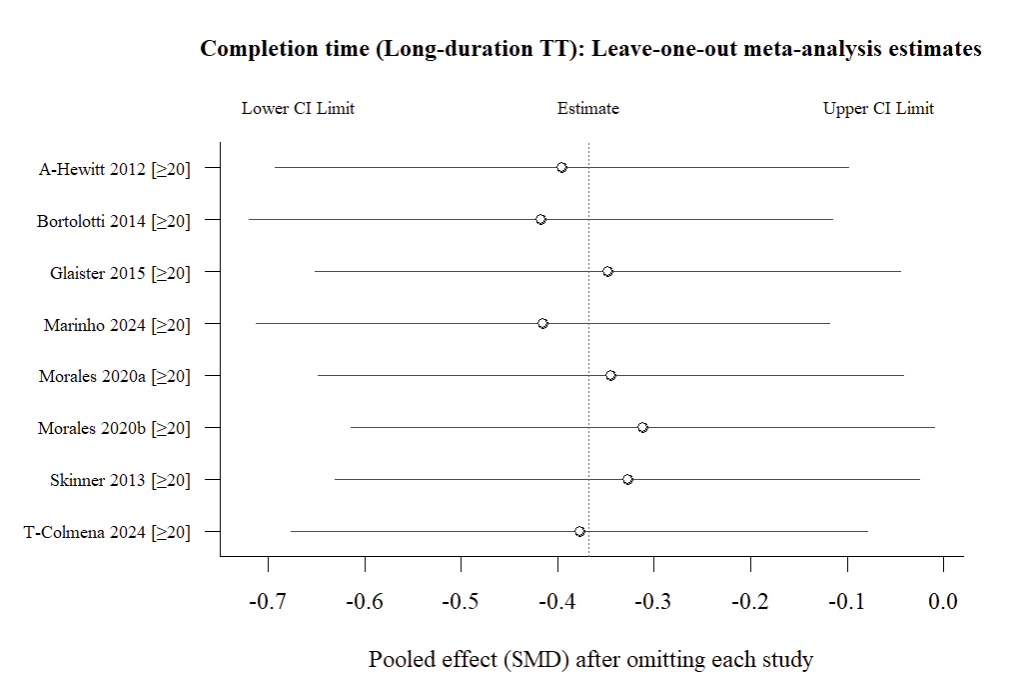


# Figure S11. Leave-one-out sensitivity analysis of completion time (long-duration TT, ≥20 min).


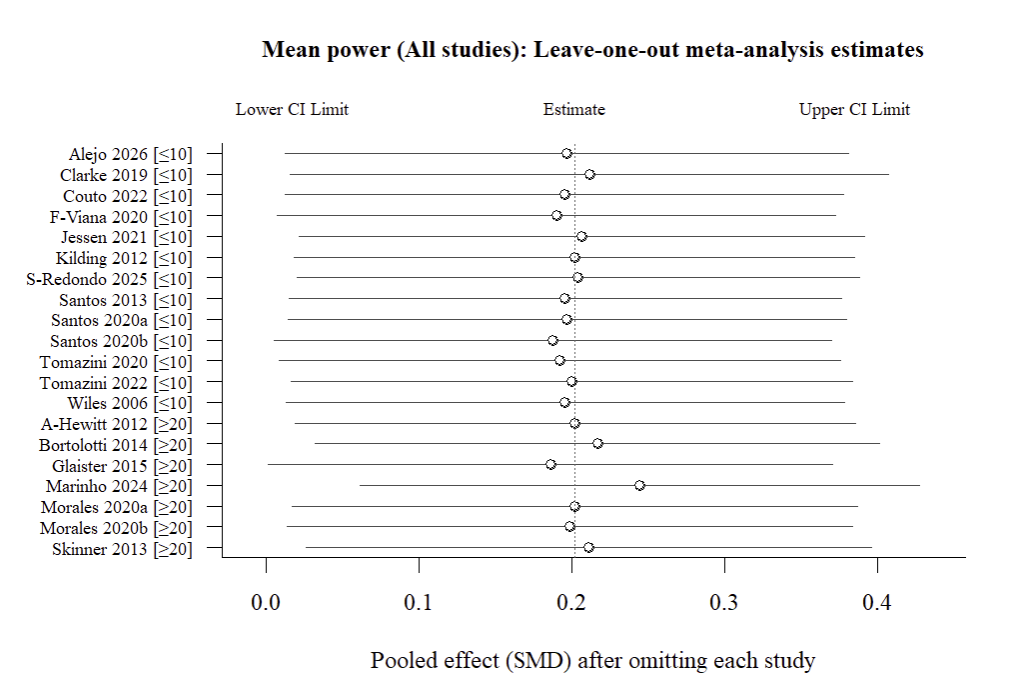


# Figure S12. Leave-one-out sensitivity analysis of mean power output (overall).


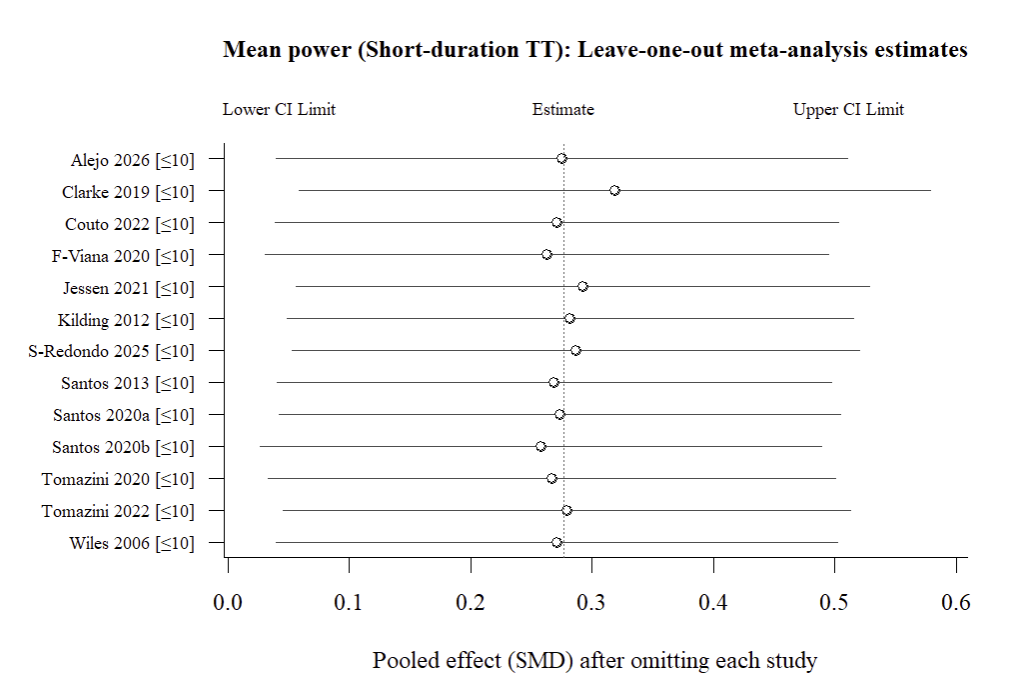


# Figure S13. Leave-one-out sensitivity analysis of mean power output (short-duration TT, ≤10 min).


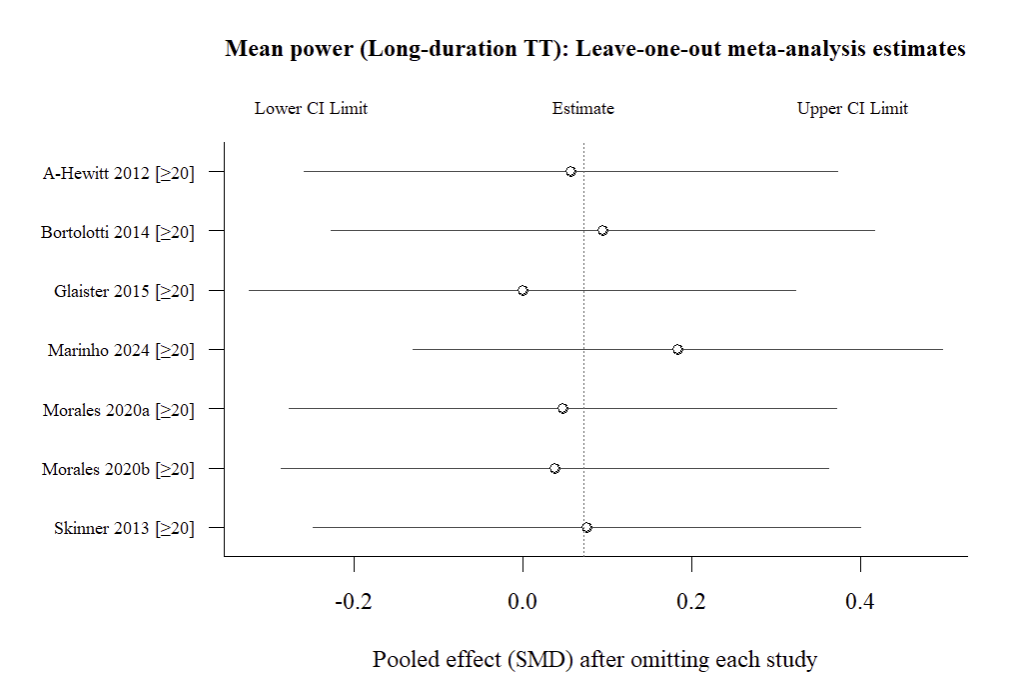


# Figure S14. Leave-one-out sensitivity analysis of mean power output (long-duration TT, ≥20 min).

# Table S1. Database Search Details

| Database | Search Strategy |
| --- | --- |
| PubMed | ("Caffeine"[Mesh] OR caffeine[tiab] OR coffee[tiab] OR caffeinated[tiab] OR gum[tiab] OR capsule*[tiab] OR pill*[tiab] OR tablet*[tiab] OR "mouth rinse"[tiab] OR solution*[tiab])  AND  ("Bicycling"[Mesh] OR cycling[tiab] OR cyclist*[tiab] OR bicycl*[tiab] OR bicycle*[tiab] OR ergometer*[tiab])  AND  (randomizedcontrolledtrial[Filter]) |
| Embase | ('caffeine'/exp OR caffeine:ti,ab OR coffee:ti,ab OR caffeinated:ti,ab OR gum:ti,ab OR capsule*:ti,ab OR pill*:ti,ab OR tablet*:ti,ab OR 'mouth rinse':ti,ab OR solution*:ti,ab)  AND  ('bicycling'/exp OR cycling:ti,ab OR cyclist*:ti,ab OR bicycl*:ti,ab OR bicycle*:ti,ab OR ergometer*:ti,ab)  AND  ('randomized controlled trial'/de) |
| Scopus | (TITLE-ABS-KEY ( caffeine OR coffee OR caffeinated ) OR TITLE-ABS-KEY (( caffeine OR coffee OR caffeinated ) PRE/3 (gum OR capsule* OR pill* OR tablet* OR "mouth rinse" OR solution*)))  AND  TITLE-ABS-KEY (bicycling OR cycling OR cyclist* OR bicycl* OR bicycle* OR ergometer* )  AND  TITLE-ABS-KEY(random* OR randomi?ed OR placebo* OR trial* OR "controlled trial" OR crossover OR "cross-over" ) |
| Cochrane Library | (  caffeine OR coffee OR caffeinated OR((gum OR capsule* OR pill* OR tablet* OR "mouth rinse" OR solution*)AND(caffeine OR coffee OR caffeinated))  )  AND  (bicycling OR cycling OR cyclist* OR bicycl* OR bicycle* OR ergometer*) |
| Web of Science | TS=(  (caffeine OR coffee OR caffeinated) OR ((gum OR capsule* OR pill* OR tablet* OR "mouth rinse" OR solution*) NEAR/3 (caffeine OR coffee OR caffeinated))  )  AND (bicycling OR cycling OR cyclist* OR bicycl* OR bicycle* OR ergometer*)  AND (random* OR placebo* OR trial* OR "controlled trial" OR crossover OR "cross-over") |

| **Table S2. Characteristics of the studies included in this meta-analysis.** | | | | | | | | | | | | | | | |
| --- | --- | --- | --- | --- | --- | --- | --- | --- | --- | --- | --- | --- | --- | --- | --- |
| Study | Country | Sample size | Age (y) | Athlete Level | Daily Caffeine Intake (mg/day) | Caffeine Dose (mg/kg) | Caffeine Intake Time (min) | Caffeine Dosage Form | Control Intervention | TT Test Completion Time (min) | Exercise mode | Cycling Test Setup | Completion Time(s) | Average Power(W) | Caffeine Withdrawal Duration |
| Acker-Hewitt et al., 2012 | United States | n=10;M | 28±9 | Trained | NR | 6 | 80 | capsule | Placebo Capsules | 49 | 20 | Laboratory | CAF：2616.0±294.0 PLA: 2652.0±270.0 | CAF: 247±47 PLA: 238±42 | 24 h before the experiment |
| Alejo et al.,2026 | Spain | n=13;NR | 16.3±0.5 | Trained | NR | 6 | 60 | NR | NR | 8 | NR | Field | NR | CAF: 363±40 PLA: 352±34 | NR |
| Bortolotti et al., 2014 | Brazil | n=13;M | 26±10 | Trained | NR | 6 | 60 | capsule | Maltodextrin Capsules | 39 | 20 | Laboratory | CAF: 2181±193.9 PLA: 2191±157.6 | CAF: 204.6±43.9 PLA: 206.9±28.5 | 48 h before the experiment |
| Carneiro et al., 2013 | Brazil | n=10;M | 27.0±8.0 | Trained | NR | 6 | 60 | capsule | Maltodextrin Capsules | 1.5 | 1 | Laboratory | CAF: 84.2±5.6 PLA: 84.8±6 | NR | 48 h before the experiment |
| Clarke et al., 2019 | United Kingdom | n = 38 M: 19 F: 19 | M: 30±5 F: 28±6 | Recreational | M: 191±118 F: 214±158 | 3 | 60 | solution | Hot Water with Coffee Flavor Essence | 9 | 5 | Laboratory | CAF: 482±51 PLA: 491±53 | CAF: 219 ± 61 PLA: 210 ± 61 | 12 h before the experiment |
| Clarke et al., 2021 | United Kingdom | n=46; 27M/19F | M:29±6 F:28±6 | Recreational | L: 153±99 H: 415±133 | 3 | 60 | solution | Isovolumetric Warm Water Solution | 8.3 | 5 | Laboratory | CAF: 482±46 PLA: 490±49 | NR | 12h before the experiment |
| Couto et al., 2022 | Brazil | n=9:M | 32.3±6.0 | Trained | 85.5±71.3 | 5 | 60 | capsule | Cellulose Capsule | 6.5 | 4 | Laboratory | CAF: 368.2±14.9 PLA: 375.1±14.5 | CAF: 323 ± 40 PLA: 308 ± 37 | 24 h before the experiment |
| Ferreira Viana, 2020 | Brazil | n=9:M | 32.0 ± 7.5 | Trained | 50–250 | 6 | 60 | capsule | Sucrose-based Capsules | 6 | 4 | Laboratory | CAF: 350.0±14.6 PLA: 357.0±13.2 | CAF: 331.4±53 PLA: 306.2±40 | 24 h before the experiment |
| Glaister M et al., 2015 | United Kingdom | n=14:F | 31±7 | Trained | 249±131 | 5 | 60 | capsule | Maltodextrin Capsules | 37 | 20 | Laboratory | CAF: 2077.2±75.6 PLA: 2122.2±102.0 | CAF: 205±21 PLA: 194±25 | 24 h before the experiment |
| Vinícius et al.,2025 | New Zealand | n=13;M | 36.2±3.3 | Trained | 458.6±375.5 | 5 | 50 | capsule | Cellulose Capsule | 6.5 | 4 | Laboratory | CAF-1: 381±10.8 CAF-2: 376±10.9 PLA: 383±17.0 | NR | NR |
| Jessen et al., 2021 | Denmark | n=14;M | 26±6 | Trained | NR | 5 | 60 | capsule | Lactose Monohydrate Capsules | 6 | NR | Laboratory | NR | CAF：317±43  PLA：312±41 | 24 h before the experiment |
| Kilding et al., 2012 | New Zealand | n=10;M | 24.2±5.4 | Trained | NR | 3 | 60 | capsule | Corn Flour Capsules | 4 | 3 | Laboratory | CAF: 226.5±9.4 PLA: 228.7±10.8 | CAF: 381±37 PLA: 373±41 | Throughout the study period |
| Marinho et al., 2024 | Brazil | n=10;M | 24.7±3.6 | Recreational | 75.44±51.51 | 5 | 90 | Capsule | Placebo Capsules + Magnesium Sulfate Mouthwash | 22 | 10 | Laboratory | CAF: 1338±323 PLA: 1321±320 | CAF: 148 ± 11 PLA: 169 ± 29 | 48 h before the experiment |
| Morales et al., 2020 | Brazil | n=14;M | 34.1±4.4 | Trained | 285.9±108.0 | 6 | 60 | capsule | Magnesium Silicate Capsules | 30 | 16 | Laboratory | CC/CP: 1634±61.29 /1692±90.47 PC/PP: 1631±90.45/1674±72.88 | CC/CP: 249.7±27.23/242.5±29.51 PC/PP: 250.3±30.24/244.1±29.33 | 24 h before the experiment |
| Sánchez-Redondo et al., 2025 | Spain | n=11;M | 17±1 | Trained | 39.5±61.1 | 3 | 60 | capsule | Maltodextrin Capsules | 8 | 8 | Field | NR | CAF: 369±31 PLA: 364±32 | 72 h before the experiment |
| Santos et al., 2020 | Brazil | n=16;M | 33.5±5.2 | Trained | NR | 5 | 60 | capsule | Cellulose Capsule | 7 | 4 | Laboratory | HP: CAF：365.5±13.1 PLA: 371.0±11.4 LP: CAF：402.5±16.1 PLA: 412.7±15.6 | HP: CAF: 329.9 ± 41.2 PLA: 316.4 ± 33.4 LP: CAF: 247.3±23.2 PLA: 232.2±21.6 | 24 h before the experiment |
| Santos et al., 2013 | Brazil | n=8;M | 32.6±5.4 | Trained | NR | 5 | 60 | capsule | Cellulose Capsule | 7 | 4 | Laboratory | CAF: 409±12 PLA: 419±13 | CAF: 232.8±21.4 PLA: 219.1±18.6 | 24 h before the experiment |
| Silva-Cavalcante et al., 2013 | Brazil | n =7;M | 32.3±5.4 | Trained | NR | 5 | 60 | capsule | Placebo Capsules | 7 | 4 | Laboratory | CAF: 404.6±17.1 PLA: 421.0±12.3 | NR | 24 h before the experiment |
| Skinner et al., 2013 | Australia | n=14;M | 31.0±5.2 | Trained | NR | 6 | 60 | capsule | Calcium Sulfate Capsules | 59.1 | 40 | Laboratory | CAF: 3475.7±97.2 PLA: 3546.2±122.8 | CAF: 255±30.9 PLA: 253.2±43 | 48h before the experiment |
| Tomazini et al., 2020 | Brazil | n=11;M | 24.5±6.9 | Recreational | 93.3±118.1 | 5 | 60 | capsule | Cellulose Capsule | 7 | 4 | Laboratory | CAF: 407.9±24.5 PLA: 416.1±34.1 | CAF: 241.4±16.1 PLA: 234.1±19.2 | 24 h before the experiment |
| Tomazini et al., 2022 | Brazil | n=11;M | 33±7 | Trained | 171±147 | 5 | 50 | capsule | Cellulose Capsule | 7 | 4 | Laboratory | OP-CAF: 367.3±15.2 OP-PLA: 370.7±14.2 | OP-CAF: 316±27.7 OP-PLA: 309.3±25.7 | 24 h before the experiment |
| Trujillo-Colmena et al.,2024 | Spain | n = 11 M: 9 F: 2 | 22±3 | Recreational | 0.79±0.64 | 3 | 60 | solution | Instant Decaffeinated Coffee Solution | 50 | 13.9 | Field | CAF: 2470.2±370.8 PLA: 2592.0±441.0 | NR | 24 h before the experiment |
| Wiles et al.,2006 | United Kingdom | n=8;M | 32±6 | Trained | NR | 5 | 60 | solution | Lemon Water Mixture Solution | 1.2 | 1 | Laboratory | CAF: 71.1±2.0 PLA: 73.4±2.3 | CAF: 523±43 PLA: 505±46 | 72 h before the experiment |

#

# Table S3. Methodological assessment of randomized controlled trials included in the systematic review using the PEDro scale.

| **Study** | **A** | **B** | **C** | **D** | **E** | **F** | **G** | **H** | **I** | **J** | **K** | **Score** |
| --- | --- | --- | --- | --- | --- | --- | --- | --- | --- | --- | --- | --- |
| Acker-Hewitt et al, 2012 | Y | 1 | 0 | 1 | 1 | 1 | 1 | 0 | 0 | 1 | 1 | 7/10 |
| Alejo et al, 2026 | Y | 1 | 1 | 1 | 1 | 1 | 1 | 1 | 1 | 1 | 1 | 10/10 |
| Bortolotti et al, 2014 | Y | 1 | 0 | 1 | 1 | 1 | 1 | 1 | 1 | 1 | 1 | 9/10 |
| Carneiro et al, 2013 | Y | 1 | 1 | 0 | 1 | 1 | 1 | 1 | 1 | 0 | 1 | 8/10 |
| Clarke et al, 2019 | Y | 1 | 0 | 1 | 1 | 1 | 1 | 1 | 1 | 1 | 1 | 9/10 |
| Clarke et al, 2021 | Y | 1 | 1 | 1 | 1 | 1 | 1 | 1 | 1 | 1 | 1 | 10/10 |
| Couto et al, 2022 | Y | 1 | 0 | 1 | 1 | 1 | 1 | 1 | 1 | 1 | 1 | 9/10 |
| Ferreira Viana et al, 2020 | Y | 1 | 0 | 1 | 1 | 1 | 1 | 1 | 1 | 1 | 1 | 9/10 |
| Glaister et al, 2015 | Y | 1 | 0 | 1 | 1 | 1 | 1 | 1 | 1 | 1 | 1 | 9/10 |
| Jessen et al, 2021 | Y | 1 | 1 | 1 | 1 | 1 | 1 | 0 | 0 | 1 | 1 | 8/10 |
| Kilding et al, 2012 | Y | 1 | 1 | 1 | 1 | 1 | 1 | 1 | 0 | 1 | 1 | 9/10 |
| Marinho et al, 2024 | Y | 1 | 1 | 1 | 1 | 1 | 1 | 1 | 1 | 1 | 1 | 10/10 |
| Morales et al, 2020 | Y | 1 | 1 | 1 | 1 | 1 | 1 | 1 | 1 | 1 | 1 | 10/10 |
| Sánchez-Redondo et al, 2025 | Y | 1 | 1 | 1 | 1 | 1 | 1 | 1 | 1 | 1 | 1 | 10/10 |
| Santos et al, 2013 | Y | 1 | 0 | 1 | 1 | 1 | 1 | 1 | 1 | 1 | 1 | 9/10 |
| Santos et al, 2020 | Y | 1 | 0 | 1 | 1 | 1 | 1 | 1 | 0 | 1 | 1 | 8/10 |
| Silva-Cavalcante et al, 2013 | Y | 1 | 1 | 1 | 1 | 1 | 1 | 1 | 1 | 1 | 1 | 10/10 |
| Skinner et al, 2013 | Y | 1 | 0 | 1 | 1 | 1 | 1 | 1 | 1 | 1 | 1 | 9/10 |
| Tomazini et al, 2020 | Y | 1 | 0 | 1 | 1 | 1 | 1 | 1 | 1 | 1 | 1 | 9/10 |
| Tomazini et al, 2022 | Y | 1 | 0 | 1 | 1 | 1 | 1 | 1 | 1 | 1 | 1 | 9/10 |
| Trujillo-Colmena et al, 2024 | Y | 1 | 0 | 1 | 1 | 1 | 1 | 1 | 1 | 1 | 1 | 9/10 |
| Vinícius et al, 2025 | Y | 1 | 1 | 1 | 1 | 1 | 1 | 1 | 1 | 1 | 1 | 10/10 |
| Wiles et al, 2006 | Y | 1 | 0 | 1 | 0 | 0 | 1 | 1 | 1 | 1 | 1 | 7/10 |

# Table S4. PRISMA 2020 checklist.

| **Section and Topic** | **Item #** | **Checklist item** | **Location where item is reported** |
| --- | --- | --- | --- |
| **TITLE** | | |  |
| Title | 1 | Identify the report as a systematic review. | Title |
| **ABSTRACT** | | |  |
| Abstract | 2 | See the PRISMA 2020 for Abstracts checklist. | Abstract |
| **INTRODUCTION** | | |  |
| Rationale | 3 | Describe the rationale for the review in the context of existing knowledge. | Introduction |
| Objectives | 4 | Provide an explicit statement of the objective(s) or question(s) the review addresses. | Introduction |
| **METHODS** | | |  |
| Eligibility criteria | 5 | Specify the inclusion and exclusion criteria for the review and how studies were grouped for the syntheses. | Methods |
| Information sources | 6 | Specify all databases, registers, websites, organisations, reference lists and other sources searched or consulted to identify studies. Specify the date when each source was last searched or consulted. | Methods |
| Search strategy | 7 | Present the full search strategies for all databases, registers and websites, including any filters and limits used. | Methods; Table S1 |
| Selection process | 8 | Specify the methods used to decide whether a study met the inclusion criteria of the review, including how many reviewers screened each record and each report retrieved, whether they worked independently, and if applicable, details of automation tools used in the process. | Methods |
| Data collection process | 9 | Specify the methods used to collect data from reports, including how many reviewers collected data from each report, whether they worked independently, any processes for obtaining or confirming data from study investigators, and if applicable, details of automation tools used in the process. | Methods |
| Data items | 10a | List and define all outcomes for which data were sought. Specify whether all results that were compatible with each outcome domain in each study were sought (e.g. for all measures, time points, analyses), and if not, the methods used to decide which results to collect. | Methods |
|  | 10b | List and define all other variables for which data were sought (e.g. participant and intervention characteristics, funding sources). Describe any assumptions made about any missing or unclear information. | Methods; Table 1; Table S2 |
| Study risk of bias assessment | 11 | Specify the methods used to assess risk of bias in the included studies, including details of the tool(s) used, how many reviewers assessed each study and whether they worked independently, and if applicable, details of automation tools used in the process. | Methods |
| Effect measures | 12 | Specify for each outcome the effect measure(s) (e.g. risk ratio, mean difference) used in the synthesis or presentation of results. | Methods |
| Synthesis methods | 13a | Describe the processes used to decide which studies were eligible for each synthesis (e.g. tabulating the study intervention characteristics and comparing against the planned groups for each synthesis (item #5)). | Methods |
|  | 13b | Describe any methods required to prepare the data for presentation or synthesis, such as handling of missing summary statistics, or data conversions. | Methods |
|  | 13c | Describe any methods used to tabulate or visually display results of individual studies and syntheses. | Methods |
|  | 13d | Describe any methods used to synthesize results and provide a rationale for the choice(s). If meta-analysis was performed, describe the model(s), method(s) to identify the presence and extent of statistical heterogeneity, and software package(s) used. | Methods |
|  | 13e | Describe any methods used to explore possible causes of heterogeneity among study results (e.g. subgroup analysis, meta-regression). | Methods |
|  | 13f | Describe any sensitivity analyses conducted to assess robustness of the synthesized results. | Methods |
| Reporting bias assessment | 14 | Describe any methods used to assess risk of bias due to missing results in a synthesis (arising from reporting biases). | Methods |
| Certainty assessment | 15 | Describe any methods used to assess certainty (or confidence) in the body of evidence for an outcome. | Methods |
| **RESULTS** | | |  |
| Study selection | 16a | Describe the results of the search and selection process, from the number of records identified in the search to the number of studies included in the review, ideally using a flow diagram. | Results |
|  | 16b | Cite studies that might appear to meet the inclusion criteria, but which were excluded, and explain why they were excluded. | Results |
| Study characteristics | 17 | Cite each included study and present its characteristics. | Results |
| Risk of bias in studies | 18 | Present assessments of risk of bias for each included study. | Results |
| Results of individual studies | 19 | For all outcomes, present, for each study: (a) summary statistics for each group (where appropriate) and (b) an effect estimate and its precision (e.g. confidence/credible interval), ideally using structured tables or plots. | Results |
| Results of syntheses | 20a | For each synthesis, briefly summarise the characteristics and risk of bias among contributing studies. | Results |
|  | 20b | Present results of all statistical syntheses conducted. If meta-analysis was done, present for each the summary estimate and its precision (e.g. confidence/credible interval) and measures of statistical heterogeneity. If comparing groups, describe the direction of the effect. | Results |
|  | 20c | Present results of all investigations of possible causes of heterogeneity among study results. | Results |
|  | 20d | Present results of all sensitivity analyses conducted to assess the robustness of the synthesized results. | Results |
| Reporting biases | 21 | Present assessments of risk of bias due to missing results (arising from reporting biases) for each synthesis assessed. | Results |
| Certainty of evidence | 22 | Present assessments of certainty (or confidence) in the body of evidence for each outcome assessed. | Results |
| **DISCUSSION** | | |  |
| Discussion | 23a | Provide a general interpretation of the results in the context of other evidence. | Discussion |
|  | 23b | Discuss any limitations of the evidence included in the review. | Discussion |
|  | 23c | Discuss any limitations of the review processes used. | Discussion |
|  | 23d | Discuss implications of the results for practice, policy, and future research. | Discussion |
| **OTHER INFORMATION** | | |  |
| Registration and protocol | 24a | Provide registration information for the review, including register name and registration number, or state that the review was not registered. | Methods |
|  | 24b | Indicate where the review protocol can be accessed, or state that a protocol was not prepared. | Methods |
|  | 24c | Describe and explain any amendments to information provided at registration or in the protocol. | - |
| Support | 25 | Describe sources of financial or non-financial support for the review, and the role of the funders or sponsors in the review. | Funding |
| Competing interests | 26 | Declare any competing interests of review authors. | Conflict of interest |
| Availability of data, code and other materials | 27 | Report which of the following are publicly available and where they can be found: template data collection forms; data extracted from included studies; data used for all analyses; analytic code; any other materials used in the review. | Data availability statement |
